# Allocation of Adult Day Care Services to Different User Groups: A Register-Based Cross-Sectional Study

**DOI:** 10.1177/11786329241231003

**Published:** 2024-02-07

**Authors:** Lisa Victoria Burrell, Hanne Marie Rostad, Tore Wentzel-Larsen, Maren Kristine Raknes Sogstad

**Affiliations:** 1 Centre for Care Research, Norwegian University of Science and Technology, Gjøvik, Norway; 2 Norwegian Centre for Violence and Traumatic Stress Studies, Oslo, Norway; 3 Centre for Child and Adolescent Mental Health, Eastern and Southern Norway, Oslo, Norway

**Keywords:** Service allocation, adult day care, dementia, intellectual disability, register data, multilevel analysis

## Abstract

The international policy of active ageing emphasises activities and social relations for long-term care recipients, for example through adult day care. Knowledge about who are allocated such services is, however, sparse. We aimed to investigate characteristics that contribute to determine allocation of adult day care for care recipients with and without dementia. This study selected all 250 687 individuals who received long-term care services on 31 December 2019 from the Norwegian Register for Primary Health Care. We added municipal level data from the Municipality-State-Reporting register and a national survey. Multilevel analyses comparing allocation of adult day care services to other services found that municipal clustering was around 20%. Care recipients who lived alone had higher odds of receiving adult day care, while the odds of receiving adult day care decreased as age increased. Disability level and gender were also significantly associated with allocation of adult day care, but in different directions for different user groups. As the unrestricted revenues of municipalities increased, the odds of allocating adult day care to people without dementia decreased. Other municipality characteristics did not significantly impact the allocation of adult day care. In conclusion, individual characteristics were more influential in allocation of adult day care than municipality characteristics, and the results uncovered clear differences between care recipients with and without dementia.

## Background

Active ageing is a policy framework that has gained international popularity over the last few decades.^1^ A main aim of this policy is to promote leisure activities and increase social involvement for older people, including those in need of long-term care.^2^ Care recipients with varying challenges should be able to use their own resources and abilities to participate in meaningful activities according to their desires and needs. Indeed, the importance of meaningful activity and social relations is evident in their ability to prevent physical disability, cognitive decline and mental ill-health.^3,4^ The policy of active ageing is closely connected to another governmental aim for long-term care services: ageing in place. Ageing in place policy states that patients should live at home in their own community for as long as possible to delay a potential transfer to a nursing home.^5^ Both of these policy frameworks require a support system with extensive resources to enable care recipients to have a meaningful and active life at home for as long as possible.^5^

Adult day care services are a part of this support system that aim to provide such meaningful activities and social companionship to home-dwelling care recipients, in line with both active ageing and ageing in place policies. In addition to activities and socialisation, adult day care services can prevent a worsening in health and delay institutional care by giving help and training in everyday activities and monitoring the health and functioning of care recipients.^6^

From 2007, after the Norwegian government presented their first action plan for services to people with dementia, dementia patients have gained increasing entry into adult day care services.^7,8^ Previous research has found that adult day care services can have positive effects for care recipients with dementia and their family caregivers.^9,10^ The large focus on adult day care for people with dementia has persisted over time, and from January 2020 the Health- and Care Services Act requires all Norwegian municipalities to provide adult day care services to people with dementia.^11^ Every individual with dementia does not have a statutory right to receive adult day care services, but the municipal health- and care services evaluate the benefit of the service based on individual needs.^12^ Another large group of users of adult day care services is people with intellectual disabilities, and the Norwegian government states that municipalities should provide adult day care services for unemployed people with intellectual disabilities.^13^ Care recipients with intellectual disabilities have historically been the main users of adult day care services, and still constitute the largest user group (37.1%).^14,15^ The content, purpose and scope of activities in adult day care services today vary since they are tailored towards these different user groups.^16^

Despite the governmental focus on active aging and ageing in place and the subsequent use of adult day care services, knowledge about who are allocated these services and why is sparse.^14^ A recent Norwegian study found large variations between municipalities in the proportion of people living at home with dementia who received adult day care services, ranging from 0% to 79%.^17^ Likewise, a Swedish study reported large municipal variation in the expenditure per user of adult day care services.^15^ The expenditure varied according to local political preferences and budget circumstances, a finding the authors described as “. . .not surprising but still disappointing” (p. 219).

Investigating municipal variation without accounting for individual variation may, however, be problematic, and information on municipal and individual factors that together determine allocation of adult day care services is lacking. Consequently, the present study aimed to investigate the individual and municipal characteristics that may contribute to determine allocation of adult day care services. Given the recent political focus on and concomitant increase of dementia patients in adult day care services, we investigated care recipients with a dementia diagnosis separately to identify potential differences in characteristics associated with service allocation. The international popularity of active ageing and ageing in place policies lend merit to the international relevance of the present study.

## Methods

This cross-sectional study investigated the associations of individual and municipal characteristics with allocation of adult day care and the amount of adult day care allocated, for care recipients with and without a dementia diagnosis.

### Data

The study merged data from 3 sources. First, we extracted IPLOS (Individual-based Nursing and Care Statistics) data from the Norwegian Register for Primary Health Care (NRPHC). This register covers all care recipients of municipal health and care services.^18^ We selected all individuals who received long-term care services on 31 December 2019, resulting in 250 687 care recipients. The dataset excluded individuals who were registered with contradicting or erroneous information, including the date of the end of a service preceding the date of service start. Secondly, we retrieved data from 2019 on the municipality where the individual received care from the KOSTRA (Municipality-State-Reporting) database. This database contains annually reported data from all Norwegian municipalities.^18^ Lastly, we included information on care models in Norwegian long-term care services, derived from a 2019 survey. This data stems from 277 municipalities (66% of Norwegian municipalities), where a municipality employee with extensive knowledge of their municipality’s long-term care services answered a questionnaire concerning the provision of long-term care services for adults. A hierarchical cluster analysis based on municipalities’ level of specialised services, use of assistive technology, and focus on disease prevention, health promotion, planning and coordination of care identified 4 different municipality clusters or models.^19^

### Dependent variables

The study investigated 2 dependent variables retrieved from the NRPHC. First, allocation of adult day care was a dichotomous variable where the comparison group consisted of recipients of other long-term care services. Second, the number of hours of adult day care per week, for those who received adult day care services, was a continuous variable.

### Independent variables

The NRPHC provided information on the individual level variables *age, gender, living arrangements* (whether individuals lived alone or not), and disability level. Healthcare personnel evaluated the disability level of care recipients in 2019 and reported data to the NRPHC. This evaluation is based on the World Health Organization’s classification of disabilities^20^ and assesses care recipients on 18 items on a scale from 1 to 5: 1 referring to no disability, 2 referring to some difficulty performing the task, but no need for assistance, and 3 or higher referring to increasing disability and need for assistance. Following the Norwegian Directorate of Health,^21^ we categorised the 18 items into 5 groups and calculated the average score in each group. The average score was calculated on item scores between 1 and 5, whereas missing scores or scores rated as ‘not relevant’ were excluded from the calculation. In total, 73.6% (184 529) of care recipients had a score between 1 and 5 on all items in all 5 groups. The group *Activities of Daily Living (ADL)* included personal hygiene, dressing, eating, using the toilet, and indoor and outdoor mobility, while *Instrumental Activities of Daily Living (IADL)* consisted of shopping, housekeeping and cooking. *Cognitive impairment* comprised memory and communication, and *Social functioning* encompassed daily decision-making, social interaction, and behavioural control. Finally, *Life management* included maintaining one’s own health and attending to one’s own finances.

The KOSTRA database provided the municipal level variables *centrality, population size*,^22^ *number of full-time equivalents (FTEs)* in the municipality’s health and care services per 10 000 inhabitants,^23^ and per capita *unrestricted revenues*.^24^ Unrestricted revenues are the finances that the municipalities have at their disposal after covering fixed costs, and hence constitutes the municipalities’ financial leeway.^25^ We classified the centrality of a municipality according to Statistics Norway’s index, which is based on travel time to workplaces and important service functions, on a scale from (1) most central to (6) least central.^26^ To ease interpretation of results, we modelled the continuous municipal level variables so that odds ratios and regression coefficients refer to the change in outcome following an increase per 10 000 inhabitants (population size), per 100 FTEs (number of FTEs), and per 10 000 Norwegian kroner (NOK) (unrestricted revenues) (10 000 NOK equals about 970 euros).

The *long-term care model* of municipalities, as derived from the hierarchical cluster analysis of survey data, describes the way health and care services are provided and can indicate municipalities’ traditions, political ideology and priorities. Four care models emerged.^19^ Municipalities classified as Care model 1 have a modest focus on planning, coordination of care, disease prevention and health promotion, few specialised services and limited use of assistive technology. This cluster consisted of 121, mostly small, municipalities. Municipalities in Care model 2 have a large focus on planning and coordination of care, disease prevention and health promotion, and 105 municipalities fitted within this model. Municipalities in Care model 3 have a focus on assistive technology, planning, and coordination of care, and consisted of 35 municipalities. Sixteen, mainly large, municipalities were classified as Care model 4, and they have a substantial focus on planning, coordination of care, disease prevention and health promotion, many specialised services and considerable use of assistive technology.

### Analysis strategy

Analyses of centrality and dispersion, frequencies, and cross tables described the variables and their relationship. We investigated the unadjusted relationships between variables by Pearson’s correlations for continuous variables, phi coefficients for categorical variables, and point-biserial correlations for correlations between continuous and categorical variables.^27^

Multilevel logistic analyses, specifically generalised mixed effects logistic models, assessed the dichotomous variable *allocation of adult day care*. In these logistic analyses, we compared adult day care services to other long-term care services in the NRPHC. Multilevel linear analyses, specifically linear mixed effects models, assessed the continuous variable *amount of adult day care*. In these linear analyses, we only included people who received adult day care services. Multilevel analyses account for clustering of outcome measures within municipalities, meaning that scores on outcome variables are not independent. Moreover, these analyses can investigate the relationship with both individual and municipal factors and assess variation in service allocation across and within municipalities.^28^ We performed separate analyses for care recipients without a dementia diagnosis and care recipients with a dementia diagnosis (registered with International Classification of Diseases, Tenth Revision, codes F00-F03 or G30, or International Classification of Primary Care, 1st or 2nd edition, code P70 in the NRPHC, IPLOS data).

Model 1 was an empty random intercept model without explanatory variables to estimate the intraclass correlation coefficient (ICC). The ICC specifies the degree of clustering, that is the degree of resemblance in allocation of adult day care services between care recipients belonging to the same municipality, and demonstrates the variation in allocation that can be attributed to the municipal level.^28^ Model 2 incorporated individual characteristics, model 3 also incorporated municipality characteristics, and model 4 additionally included the long-term care model of municipalities. The likelihood ratio test assessed potential improvements in predictive power from one model to the next by considering the change in deviance and degrees of freedom.^28^

We constructed the dataset in R (version 4.1.2)^29^ by using the data.table package^30^ and estimated multilevel models with the glmmTMB package.^31^

### Ethical approvals

Gaining access to the 3 different data sources required different ethical approvals. First, data from KOSTRA is publicly available and do not require an ethical approval. Second, we received register data from the NRPHC after the Regional Committee for Medical and Health Research Ethics in Norway gave an exemption to the requirement of informed consent (reference number 76190). Third, the Norwegian Centre for Research Data approved the web-based survey study that forms the basis of the long-term care models (reference number 847216). Information about the survey was attached to the email sent to potential respondents, and the email text stated that by completing the survey, the person consented to participation. Since the survey study is not classified as medical and health research (research on humans, human biological material or personal health information that aims to generate new knowledge about health and diseases), it does not require an ethical approval according to the Norwegian Act on Medical and Health Research.^32^ We performed the study following the ethical standards in the 1964 Declaration of Helsinki and its amendments.

## Results

### Descriptive statistics

Of the 250 687 care recipients in the dataset, 23 887 received adult day care services per 31 December 2019. Moreover, 92.50% of these adult day care recipients had a registered number of hours per week, with a mean of 13.94 (SD: 10.27). The care recipients who received adult day care were also likely to receive home care (69.86%) and practical assistance (55.77%). Furthermore, 16.55% (2784) of recipients of adult day care had a dementia diagnosis. Compared to care recipients without dementia, care recipients with dementia received fewer hours of care (mean: 12.10, SD: 7.71 vs mean: 15.57, SD: 11.10).

Table 1 presents the characteristics of recipients of adult day care services. The majority of care recipients were women, and the mean age of care recipients with dementia was substantially higher than care recipients without dementia. Moreover, the majority of care recipients lived alone, especially those without dementia. The mean disability level was particularly high for life management and IADL and did not substantially differ between care recipients with and without dementia.

**Table 1.** Characteristics of recipients of adult day care services.

N (%)^a^	Care recipients *with* dementia	Care recipients *without* dementia
Gender		
Female	1732 (62.26)	8129 (57.91)
Male	1050 (37.74)	5909 (42.09)
Living arrangements		
Living alone	1340 (52.00)	9492 (71.26)
Cohabiting	1237 (48.00)	3828 (28.74)
Municipalities’ long-term care model		
Care model 1	375 (16.40)	1882 (17.36)
Care model 2	1188 (51.97)	5134 (47.35)
Care model 3	342 (14.96)	1743 (16.08)
Care model 4	381 (16.67)	2083 (19.21)
Mean (SD)^b^		
Age	81.46 (8.41)	61.06 (24.44)
ADL	2.15 (0.84)	2.33 (1.05)
IADL	3.48 (0.98)	3.39 (1.12)
Cognitive impairment	2.70 (0.78)	2.31 (1.05)
Social functioning	2.47 (0.80)	2.67 (1.14)
Life management	3.55 (0.96)	3.45 (1.22)
Centrality (1: most central; 6: least central)	2.88 (1.34)	2.89 (1.29)
Population size	136 172.27 (216 792.42)	106 737.95 (182 196.51)
Unrestricted revenues per inhabitant	58 175.10 (6 224.80)	57 866.67 (6 101.53)
Number of FTEs per 10 000 inhabitants	309.28 (85.11)	313.15 (90.77)

Abbreviations: ADL, activities of daily living; IADL, instrumental activities of daily living; FTEs, full-time equivalents.

a N (%) for categorical variables.

b Mean and standard deviation (SD) for continuous variables.

### Correlations – unadjusted relationships

Table 2 presents correlations between study variables for people who receive adult day care services. Care recipients with dementia received less hours of adult day care than care recipients without a dementia diagnosis. The significant positive correlations between disability level and hours of care show that care recipients with greater disability receive more hours of adult day care. Moreover, the significant negative correlation between age and hours of care revealed that younger care recipients receive more care. Men receive more adult day care, whereas living arrangement was not significantly correlated with hours of care. Analyses also found significant, albeit small, correlations between municipality characteristics and hours of adult day care.

**Table 2.** Correlations of the amount of adult day care services with individual and municipality characteristics.

	Variable	1.	2.	3.	4.	5.	6.	7.	8.	9.	10.	11.	12.	13.	14.
1.	Amount of adult day care	1													
2.	Dementia diagnosis (0 = no/1 = yes)	−0.118^‡,b^	1												
3.	ADL	0.225^‡^	−0.063^‡,b^	1											
4.	IADL	0.307^‡^	0.029^‡,b^	0.790^‡^	1										
5.	Cognitive impairment	0.338^‡^	0.138^‡,b^	0.593^‡^	0.685^‡^	1									
6.	Social functioning	0.424^‡^	−0.067^‡,b^	0.577^‡^	0.676^‡^	0.773^‡^	1								
7.	Life management	0.400^‡^	0.031^‡,b^	0.606^‡^	0.770^‡^	0.746^‡^	0.797^‡^	1							
8.	Age	−0.441^‡^	0.318^‡,b^	−0.150^‡^	−0.176^‡^	−0.318^‡^	−0.553^‡^	−0.398^‡^	1						
9.	Gender (0 = female/1 = male)	0.141^‡,b^	−0.033^‡,a^	0.080^‡,b^	0.159^‡,b^	0.163^‡,b^	0.194^‡,b^	0.182^‡,b^	−0.207^‡,b^	1					
10.	Living arrangements (0 = cohabitant/1 = alone)	−0.012^b^	−0.152^‡,a^	−0.033^‡,b^	−0.070^‡,b^	−0.095^‡,b^	−0.038^‡,b^	−0.050^‡,b^	0.108^‡,b^	−0.158^‡,a^	1				
11.	Centrality (1: most central; 6: least central)	−0.041^‡^	−0.002^b^	−0.075^‡^	−0.051^‡^	−0.021^†^	0.012	−0.002	−0.041^‡^	0.001^b^	0.041^‡,b^	1			
12.	Population size	0.117^‡^	0.058^‡,b^	0.118^‡^	0.114^‡^	0.075^‡^	0.031^‡^	0.050^‡^	0.061^‡^	−0.001^b^	−0.010^b^	−0.593^‡^	1		
13.	Unrestricted revenues	0.030^‡^	0.019*,^b^	0.004	0.010	0.014*	−0.010	0.002	0.060^‡^	−0.009^b^	0.018^†,b^	0.321^‡^	0.247^‡^	1	
14.	Number of FTEs	−0.046^‡^	−0.016*,^b^	−0.061^‡^	−0.044^‡^	−0.005	0.024^‡^	0.003	−0.044^‡^	0.010^b^	0.029^‡,b^	0.692^‡^	−0.464^‡^	0.394^‡^	1

a Phi coefficient.

b Point-biserial correlation.

* *P* < .05, ^†^*P* < .01, ^‡^*P* < .001.

Different individual characteristics were significantly correlated, with particularly strong positive correlations between the different types of disability. The significant negative correlations between age and disability level across all types of disability demonstrate that younger care recipients have a greater disability level. Younger care recipients more seldom live alone and have a lower occurrence of dementia diagnoses. Moreover, strong correlations between municipality characteristics showed that municipalities with more inhabitants were more central and had less FTEs in the municipalities’ long-term care sector relative to their population size. Finally, more central municipalities had less unrestricted revenues.

### Multilevel analyses

Table 3 presents the results for allocation of adult day care, and Table 4 the results for the amount of adult day care. The ICC for allocation of adult day care services was 0.261 for care recipients with dementia and 0.210 for care recipients without dementia. The ICCs for the number of hours of adult day care allocated was 0.209 and 0.217, respectively. Consequently, more than 20% of the total variation in the number of hours of adult day care allocated to people with and without a dementia diagnosis was at the municipal level.

**Table 3.** Multilevel logistic generalised mixed effects models for adult day care services.

	Model 2. Individual characteristics				Model 3. Municipality characteristics				Model 4. Municipality care model			
	Care recipients *with* dementia		Care recipients *without* dementia		Care recipients *with* dementia		Care recipients *without* dementia		Care recipients *with* dementia		Care recipients *without* dementia	
Fixed effect	OR (95% CI)	*P*	OR (95% CI)	*P*	OR (95% CI)	*P*	OR (95% CI)	*P*	OR (95% CI)	*P*	OR (95% CI)	*P*
Individual characteristics												
ADL^a^	0.52 (0.48; 0.56)	<.001	0.75 (0.73; 0.78)	<.001	0.52 (0.48; 0.56)	<.001	0.75 (0.73; 0.78)	<.001	0.53 (0.49; 0.58)	<.001	0.77 (0.74; 0.80)	<.001
IADL^a^	1.03 (0.95; 1.12)	.495	1.30 (1.26; 1.34)	<.001	1.03 (0.95; 1.12)	.479	1.30 (1.26; 1.34)	<.001	1.06 (0.97; 1.16)	.225	1.31 (1.26; 1.36)	<.001
Cognitive impairment^a^	1.13 (1.03; 1.24)	.010	1.24 (1.20; 1.28)	<.001	1.13 (1.03; 1.24)	.009	1.24 (1.20; 1.28)	<.001	1.09 (0.99; 1.20)	.088	1.22 (1.18; 1.27)	<.001
Social functioning^a^	0.73 (0.66; 0.80)	<.001	1.14 (1.10; 1.18)	<.001	0.73 (0.66; 0.80)	<.001	1.14 (1.10; 1.18)	<.001	0.72 (0.65; 0.81)	<.001	1.13 (1.09; 1.18)	<.001
Life management^a^	0.83 (0.77; 0.90)	<.001	1.18 (1.15; 1.22)	<.001	0.83 (0.77; 0.91)	<.001	1.18 (1.15; 1.22)	<.001	0.83 (0.76; 0.91)	<.001	1.20 (1.16; 1.24)	<.001
Age	0.981 (0.975; 0.987)	<.001	0.992 (0.991; 0.993)	<.001	0.981 (0.975; 0.987)	<.001	0.992 (0.991; 0.993)	<.001	0.98 (0.97; 0.99)	<.001	0.993 (0.992; 0.994)	<.001
Gender (0 = female/1 = male)	1.33 (1.21; 1.48)	<.001	0.79 (0.76; 0.83)	<.001	1.34 (1.21; 1.48)	<.001	0.79 (0.76; 0.83)	<.001	1.36 (1.21; 1.52)	<.001	0.80 (0.77; 0.84)	<.001
Living arrangements (0 = cohabitant/1 = alone)	1.35 (1.22; 1.49)	<.001	1.94 (1.86; 2.03)	<.001	1.35 (1.22; 1.49)	<.001	1.94 (1.86; 2.03)	<.001	1.40 (1.25; 1.56)	<.001	1.97 (1.87; 2.07)	<.001
Municipality characteristics												
Centrality (1: most central; 6: least central)					1.07 (0.87; 1.32)	.503	0.96 (0.84; 1.10)	.571	1.07 (0.86; 1.33)	.554	0.98 (0.85; 1.14)	.834
Population size (per 1000 inhabitants)					1.01 (0.97; 1.04)	.701	1.00 (0.98; 1.03)	.793	1.01 (0.98; 1.04)	.521	1.01 (0.98; 1.03)	.547
Unrestricted revenues (per 10 000 NOK)					0.70 (0.53; 0.91)	.009	0.66 (0.56; 0.77)	<.001	0.79 (0.58; 1.08)	.135	0.67 (0.55; 0.82)	<.001
Number of FTEs (per 100 FTEs)					0.99 (0.82; 1.20)	.932	1.09 (0.97; 1.23)	.160	0.91 (0.75; 1.12)	.377	1.07 (0.94; 1.22)	.335
Municipalities’ long-term care model												
Care model 1									Ref.		Ref.	
Care model 2									0.81 (0.55; 1.19)	.279	0.96 (0.74; 1.24)	.738
Care model 3									1.07 (0.65; 1.77)	.793	0.99 (0.69; 1.40)	.935
Care model 4									0.87 (0.44; 1.72)	.692	0.78 (0.48; 1.27)	.324
Random effect	Variance component		Variance component		Variance component		Variance component		Variance component		Variance component	
Municipal level variance	1.37 (1.17)		0.92 (0.96)		1.38 (1.17)		0.82 (0.91)		1.07 (1.03)		0.68 (0.83)	

a Scale 1: very good; 5: very poor.

**Table 4.** Multilevel linear mixed effects models for the amount of adult day care services.

	Model 2. Individual characteristics				Model 3. Municipality characteristics				Model 4. Municipality care model			
	Care recipients *with* dementia		Care recipients *without* dementia		Care recipients *with* dementia		Care recipients *without* dementia		Care recipients *with* dementia		Care recipients *without* dementia	
Fixed effect	Estimate (95% CI)	*P*	Estimate (95% CI)	*P*	Estimate (95% CI)	*P*	Estimate (95% CI)	P	Estimate (95% CI)	*P*	Estimate (95% CI)	*P*
Individual characteristics												
ADL^a^	−0.51 (−1.01; −0.01)	.046	−0.93 (−1.18; −0.68)	<.001	−0.51 (−1.02; −0.01)	.044	−0.93 (−1.18; −0.68)	<.001	−0.65 (−1.22; −0.08)	.025	−1.05 (−1.35; −0.76)	<.001
IADL^a^	0.41 (−0.12; 0.93)	.126	1.28 (0.98; 1.58)	<.001	0.41 (−0.11; 0.94)	.124	1.28 (0.98; 1.58)	<.001	0.55 (−0.05; 1.14)	.072	1.34 (0.99; 1.69)	<.001
Cognitive impairment^a^	0.39 (−0.18; 0.97)	.183	0.37 (0.11; 0.64)	.006	0.39 (−0.19; 0.97)	.185	0.37 (0.11; 0.64)	.006	0.41 (−0.25; 1.07)	.220	0.32 (0.02; 0.63)	.038
Social functioning^a^	0.11 (−0.51; 0.73)	.724	0.35 (0.04; 0.65)	.026	0.12 (−0.50; 0.74)	.710	0.36 (0.05; 0.66)	.023	0.03 (−0.66; 0.73)	.926	0.44 (0.09; 0.79)	.015
Life management^a^	0.76 (0.26; 1.26)	.003	1.06 (0.80; 1.33)	<.001	0.76 (0.26; 1.26)	.003	1.06 (0.80; 1.33)	<.001	0.79 (0.23; 1.35)	.006	1.17 (0.87; 1.47)	<.001
Age	−0.05 (−0.09; −0.01)	.014	−0.17 (−0.18; −0.16)	<.001	−0.05 (−0.09; −0.01)	.015	−0.17 (−0.18; −0.16)	<.001	−0.04 (−0.08; 0.01)	.099	−0.18 (−0.19; −0.16)	<.001
Gender (0 = female/1 = male)	0.96 (0.33; 1.58)	.003	0.35 (0.03; 0.68)	.034	0.94 (0.32; 1.56)	.003	0.36 (0.03; 0.68)	.032	0.88 (0.17; 1.59)	.015	0.42 (0.04; 0.80)	.032
Living arrangements (0 = cohabitant/1 = alone)	1.63 (1.01; 2.25)	<.001	0.33 (−0.03; 0.69)	.069	1.62 (1.00; 2.25)	<.001	0.33 (−0.02; 0.69)	.067	1.60 (0.88; 2.32)	<.001	0.38 (−0.04; 0.79)	.075
Municipality characteristics												
Centrality (1: most central; 6: least central)					−0.55 (−1.37; 0.27)	.190	0.13 (−0.51; 0.77)	.689	−0.07 (−1.06; 0.92)	.896	0.34 (−0.43; 1.10)	.385
Population size (per 1000 inhabitants)					0.00 (−0.10; 0.11)	.936	0.03 (−0.07; 0.14)	.536	0.02 (−0.10; 0.14)	.729	0.06 (−0.05; 0.17)	.280
Unrestricted revenues (per 10 000 NOK)					0.54 (−0.77; 1.85)	.417	0.17 (−0.72; 1.07)	.706	0.35 (−1.24; 1.95)	.665	−0.41 (−1.57; 0.76)	.497
Number of FTEs (per 100 FTEs)					0.01 (−0.80; 0.82)	.977	−0.61 (−1.20; −0.01)	.046	−0.26 (−1.19; 0.68)	.593	−0.51 (−1.17; 0.16)	.140
Municipalities’ long-term care model												
Care model 1									Ref.		Ref.	
Care model 2									1.20 (−0.65; 3.05)	.204	0.52 (−0.80; 1.84)	.440
Care model 3									0.51 (−1.78; 2.81)	.661	1.36 (−0.38; 3.09)	.126
Care model 4									−0.26 (−3.27; 2.75)	.865	−1.61 (−3.96; 0.74)	.180
Random effect	Variance component		Variance component		Variance component		Variance component		Variance component		Variance component	
Municipal level variance	13.24 (3.64)		13.73 (3.71)		12.78 (3.58)		13.38 (3.66)		14.28 (3.78)		12.92 (3.60)	
Individual level variance	49.30 (7.02)		79.65 (8.93)		49.34 (7.02)		79.66 (8.93)		52.59 (7.25)		82.98 (9.11)	

a Scale 1: very good; 5: very poor.

Model 2 included individual characteristics, and the likelihood ratio tests between the first and second models were significant (*P* < .001 for all tests). Model 3 included municipality characteristics, and the likelihood ratio tests between the second and third models were significant for allocation of adult day care services (*P* = .007 for care recipients with dementia, *P* < .001 for care recipients without dementia), but not for the number of hours allocated (*P* = .623 for care recipients with dementia, *P* = .252 for care recipients without dementia). Finally, model 4 included information about the long-term care model of the municipality, and the likelihood ratio tests between the 3rd and 4th models were not significant (adult day care: *P* = .620 for care recipients with dementia, *P* = .801 for care recipients without dementia; amount of adult day care: *P* = .542 for care recipients with dementia, *P* = .121 for care recipients without dementia).

For care recipients with dementia, greater disability levels for ADL, social functioning and life management were significantly associated with lower odds of receiving adult day care services. For care recipients without dementia, however, greater disability levels for IADL, cognitive impairment, social functioning and life management were associated with greater odds of allocation, but a greater ADL disability was associated with lower odds of allocation. When investigating the amount of adult day care services, the disability level of care recipients with dementia was not very influential for service allocation: only a significant negative association with ADL and a significant positive association with life management appeared. For care recipients without dementia, however, increased disability for IADL, cognitive impairment, social functioning and life management were positively associated with the number of hours allocated, while increased ADL disability was negatively associated with the amount of care.

Furthermore, care recipients with and without dementia had lower odds of being allocated adult day care services the older they became. Moreover, care recipients without dementia were allocated less hours the older they became. Both groups also had higher odds of being allocated adult day care services if they lived alone, and care recipients with dementia received significantly more care if they lived alone. Men with dementia and women without dementia had higher odds of receiving adult day care compared to the opposite sex. Moreover, men received more hours of adult day care regardless of diagnosis.

The only significant association between municipality characteristics and service allocation revealed that care recipients without dementia had lower odds of being allocated adult day care in municipalities with more unrestricted revenues. No significant associations appeared between centrality, population size, number of FTEs or municipalities’ long-term care models and service allocation.

## Discussion

The multitude of study results presented above can be shortly summarised into the following 2 points. First, individual characteristics were more influential in predicting allocation of adult day care than the structure and care model of municipalities. Second, the characteristics predicting service allocation differed between care recipients with and without dementia. The discussion will summarise study results in more detail, compare them to previous studies and provide possible explanations.

### Similarities between municipalities – municipality characteristics

Municipal clustering in the number of hours of adult day care services allocated was slightly above 20%, which is considerably higher than for home-based services (3.5%).^33^ However, except for a significant negative association between unrestricted revenues and allocation of adult day care to care recipients without dementia, the structure and care model of municipalities did not significantly impact the allocation of adult day care. Future research should seek to identify the degree of municipal clustering in allocation of other long-term care services to increase transparency in the allocation process.

As opposed to municipality characteristics, the impact of individual characteristics was often significant with larger effects. This is in line with the Norwegian government’s strategic plans for the health and care sector that highlight the importance of individual needs and preferences in the allocation of long-term care services.^34,35^ The limited influence of municipality characteristics compared to individual characteristics is consistent with a similar Norwegian study investigating allocation of home-based services and long-term stay in institutions.^33^ Consequently, future studies that investigate municipal variation should include individual characteristics given their large influence and diversity across municipalities.

The limited influence of municipality characteristics can be caused by a development towards diminished municipal variability and reduced leeway in service allocation.^36^ Indeed, the differences in economic conditions and performance between Norwegian municipalities have decreased since 2013.^37^ Combined with an increase in government regulations and a tightening of overall resources, each municipality’s flexibility in developing and allocating services has become constrained.^38^ In addition, the reduced variation between municipalities can be a result of benchmarking: municipalities trying to adapt to the allocation level of other municipalities by comparing publicly available municipal data.^36^

The significant negative association between unrestricted revenues and allocation of adult day care for care recipients without dementia indicates that municipalities with a better economy more often allocate other long-term care services to care recipients without dementia, typically people with intellectual disabilities, physical limitations and psychiatric disorders.^14^ Indeed, a previous Norwegian study using the NRPHC found that municipalities with higher unrestricted revenues allocate more long-term institutional stays.^33^ A main reason for this probably stems from the lower costs of adult day care services compared to other long-term care services, especially institutional care.^6^ Such unwanted municipal variation based on differing resources should be further investigated to ensure equal and just allocation of services.

For people with dementia, however, unrestricted revenues were not significantly associated with service allocation. Given that all Norwegian municipalities were required to provide adult day care services for people with dementia from January 2020,^11^ the data from December 2019 that was used in the present study would reflect this increased focus on dementia patients. In addition to this judicial incentive, economic subsidisation guide municipalities towards establishing and sustaining adult day care services for dementia patients.^6^

### Differences between diagnoses – individual characteristics

Contrary to municipality characteristics, individual characteristics greatly influenced adult day care allocation. Additionally, the impact of individual characteristics differed between care recipients with and without a dementia diagnosis. Despite a similar disability level, care recipients with dementia were substantially older than those without a dementia diagnosis and received fewer hours of care. For care recipients without dementia, younger people received more hours of care. Clearly, age is a governing factor in the allocation of adult day care, and younger care recipients gain priority regardless of disability level. Age is also influential in allocation of home-based services, where a previous study reported that younger care recipients received more hours of care.^33^ Older care recipients are, however, more likely to be allocated a long-term institutional stay.^39^

Younger people with extensive needs are, hence, the primary focus for home-based rehabilitation, reablement and active ageing measures. Romøren^40^ characterised this as different ‘care regimes’ for younger and older care recipients. Since younger recipients of adult day care services often have intellectual disabilities,^14^ the differences between age groups can also indicate that people with intellectual disabilities are prioritised. These results are in line with previous findings demonstrating that people with intellectual disabilities receive more practical assistance compared to care recipients with a comparable disability level who are diagnosed with somatic disorders, mental disorders or dementia.^40^

Care recipients’ living arrangements also influenced service allocation. Care recipients who lived alone were more likely to receive adult day care services regardless of diagnosis, and care recipients with dementia who lived alone received more hours of care. Previous studies have also reported that care recipients who lived alone received more home-based services,^41-43^ and these findings probably stem from a lacking proximity to close family who often contribute with practical and medical help, socialisation and activation.^44^ Additionally, analyses uncovered gender differences: Compared to the opposite sex, men with dementia and women without dementia had higher odds of receiving adult day care.

In addition to age, living arrangements and gender, the disability level of care recipients was significantly associated with service allocation. Importantly, the associations between disability level and adult day care were weaker for care recipients with dementia compared to care recipients without dementia. This difference between diagnoses may indicate that other factors related to individual preferences, political focus, judicial regulations and economic support play a larger role in allocation of adult day care services for dementia patients. Uncovering the specific mechanisms involved should be a focus for future research. Increased disability was associated with a decreased likelihood of adult day care services compared to other services for care recipients with dementia, but the opposite was true for care recipients without dementia. The decreased likelihood of adult day care services for highly disabled and elderly dementia patients can probably be attributed to a higher chance of long-term institutional stays for this group.^33^ Again, we see that younger people with intellectual disability and psychiatric disorders are a priority for home-based rehabilitation, reablement, socialisation and activation.^40^

### Strengths and limitations

The present study is the first to investigate the associations of both individual and municipal characteristics with allocation of adult day care services, as well as novel in its comparison of the different user groups. The national coverage of the NRPHC and KOSTRA registers and the representative sample of municipalities in the survey study contribute to a high external validity.

As with all register studies, the recorded data may not perfectly reflect actual practice. Healthcare workers may digress from what is stated in the official allocation letter^45^ by offering services to people who have not been granted that service. Alternatively, care recipients may not use the service, or may use fewer hours of care than what they are assigned. IPLOS data in the NRPHC has relatively high quality, with a recent increase in the reporting of patient diagnoses.^46^ However, only 44% of care recipients in 2017 had a registered diagnosis.^46^ We can, as a result, postulate that care recipients with beginning dementia are not registered with a diagnosis, but rather included in the category of people without a dementia diagnosis. The same underdiagnosing may occur for elderly who develop dementia in nursing homes. These misclassifications increase the chance of a type II error.

## Conclusions

Individual characteristics were more influential in allocation of adult day care services than municipality characteristics, and the study results uncovered clear differences between care recipients with and without dementia. Other factors related to individual preferences, political focus, judicial regulations, and economic support may be more influential in allocation of adult day care services for care recipients with dementia. Transparency in the allocation process and service distribution based on individual needs and circumstances is a prerequisite for fairly distributed universal services.
